# The role of community health workers in the management of hypertension in Nigeria

**DOI:** 10.1186/s12875-024-02521-2

**Published:** 2024-07-20

**Authors:** Tijani Idris Ahmad Oseni, Abdulgafar Lekan Olawumi, Tawakalit Olubukola Salam, Amudalat Issa, Mohammed Abubakar Abiso, Ibraheem Sanusi, Temitope Ilori

**Affiliations:** 1 Department of Family Medicine, Edo State University, Uzairue, Nigeria; 2 Department of Family Medicine, Irrua Specialist Teaching Hospital, Irrua, Nigeria; 3 Department of Family Medicine, Aminu Kano Teaching hospital, Kano, Nigeria; 4 Department of Family Medicine, University College Hospital, Ibadan, Nigeria; 5 Children Specialist Hospital, Ilorin, Nigeria; 6 Department of Family Medicine, University of Maiduguri Teaching Hospital, Maiduguri, Nigeria; 7 Kwara State Specialist Hospital, Sobi, Nigeria; 8 Family Medicine Unit, Department of Community Medicine, University of Ibadan, Ibadan, Nigeria

**Keywords:** Community Health workers, Hypertension management, Non-communicable diseases, Nigeria, Hypertensive patients, Primary healthcare centre

## Abstract

**Background:**

Hypertension is the number one risk factor for cardiovascular death worldwide and its prevalence has been on the increase in LMICs including Nigeria. There is an increasing awareness and recognition of the contributions of the community health workers (CHWs) in the healthcare system. This study assessed their current role in the management of hypertension and patient satisfaction with the care received.

**Methods:**

A mixed method study (cross-sectional study of 381 CHWs and key informant interview of 14 patients with hypertension selected using multi-stage and purposive sampling respectively) was conducted across five states in different geopolitical zones of Nigeria to assess the role of CHWs in hypertension management and the patients’ level of satisfaction with services of CHWs. Chi-square test was used to assess relationship between categorical variables. A p-value ≤ 0.05 was considered statistically significant. Thematic analysis of the text data from the KII was done using Nvivo^®^ version 12 pro.

**Results:**

A total of 381 CHWs completed the study. They were predominantly males (63%) with mean age of 40.96 ± 12.51 years. Only about one-third of the CHWs (31%) could correctly diagnose hypertension while only 15% knew the base-line investigations to be requested. Being female (FE = 9.205, *p* = 0.008) and resident in northwest geopolitical region (χ^2^ = 20.920, *p* < 0.001) had statistically significant associations with appropriate diagnostic skills for hypertension. Being supervised by doctors was associated with appropriate knowledge of baseline investigations for hypertension (χ^2^ = 5.534, *p* = 0.019). Mostly, hypertensive patients reported positive experiences and satisfaction with the services provided by the CHWs.

**Conclusions:**

Community health workers currently have critical contributions in the management of hypertension in Nigeria. Hypertensive patients generally reported satisfactory experience with CHWs managing them. The services rendered by CHWs can be improved upon by adequate supervision and training.

**Supplementary Information:**

The online version contains supplementary material available at 10.1186/s12875-024-02521-2.

## Introduction

Globally, hypertension is the leading cause of cardiovascular morbidity and mortality. It is defined as systolic blood pressure (SBP) ≥ 140 mmHg and/or diastolic blood pressure (DBP) ≥ 90 mmHg [1, 2]. Between 1990 and 2019, the number of adults aged 30–79 years living with hypertension globally increased from 650 million to 1.28 billion, primarily due to population growth and ageing [3]. However, there is a disparity in hypertension prevalence trends between high-income countries (HICs) and low- and middle-income countries (LMICs). While the prevalence of hypertension is reducing in HICs, many LMICs are experiencing an increasing prevalence of hypertension with a recent study showing a hypertension prevalence of 38.1% in Nigeria [2] with some parts of the country having a prevalence of 60% [4]. This is largely due to the high consumption of unhealthy diets in LMICs as compared to HICs. This could be because foods like vegetables, fruits, and nuts are not readily available and affordable in LMICs [5].

Health service delivery is largely dependent on professional health workers such as doctors and nurses, providing optimal and quality care to patients. The workforce is the backbone of every health system and is essential to improving health [6]. However, effective management of hypertension requires a multifaceted approach, including community-based interventions [7]. The burden of hypertension in Nigeria may increase further in years to come due to the increasing adult population and the changing lifestyle of Nigerians [8]. This may overstretch the already frail healthcare system. Community health workers may play a crucial role in expanding access to basic healthcare services, particularly in remote areas, and helping to close the health equality gap [9].

Compared to professional healthcare workers like nurses and doctors, community health workers are healthcare providers who reside in the community they serve and have less formal education and training [9]. The vital role that community health workers play in many nations has come to light in recent years, and their contribution to various health programmes is now greatly appreciated [10]. These human resources have a great potential to provide healthcare services to underserved populations, like marginalized groups and communities living in remote areas. They provide health services in a culturally appropriate manner, increase access to services, address disparities in health status, and enhance the effectiveness and performance of the health system [9].

In Nigeria, community health workers (CHWs) are the Community Health Officers (CHOs), Community Health Extension Workers (CHEWs), and Junior Community Health Extension Workers (JCHEWs). Community Health Officers receive the highest level of training and are based at health facilities and provide a broad range of primary health care services. They oversee CHEWs and JCHEWs, who work at health facilities and in communities. The Federal Ministry of Health employ all the three cadres [11]. They provide a wide range of services including maternal and child health, family planning, malaria, HIV and AIDS, with less involvement in the prevention and control of non-communicable diseases. Their services have evolved over time, from health promotion and prevention to more supportive functions that are connected to the rising prevalence of chronic diseases such as hypertension and diabetes [12]. Deploying CHWs for awareness, screening, and prevention activities is paramount in hypertension care, especially since they are close to the people and are familiar with them [13].

The roles and responsibilities of CHWs in hypertension management encompass a wide range of tasks, including but not limited to screening, education, lifestyle counselling, medication adherence support, and linkage to healthcare services. Their unique position within the communities enables them to deliver culturally appropriate and acceptable care, thereby addressing barriers to healthcare access and improving health outcomes among underserved populations [14].

Evidence shows that CHWs often go beyond their mandated roles outlined in the National standing orders. This suggests that with proper training and supervision, they can be empowered to assume greater responsibilities in hypertension management, thus enhancing the capacity of the healthcare workforce [15]. Despite the growing recognition of their contributions, there remains a need for a comprehensive understanding of the specific roles and responsibilities of CHWs in hypertension management [16]. In this study, the primary objectives were to assess the current role of CHWs in the management of hypertension in Nigeria and to assess patient satisfaction with the treatment they receive from CHWs. Additionally, the research also aimed at identifying the need for training and supervision of CHWs.

## Methods

### Study design

Mixed method (Cross-sectional study of the CHW and in-depth interview of hypertensive patients assessing care from the CHWs).

### Study sites

Primary Healthcare Centres (PHC) in Kano, Borno, Kwara, Oyo, and Edo state.

### Study population

Community Health Workers (CHWs).

### Sample size determination

The total number of healthcare workers in Nigeria is 347,052 [17].

The estimated population of CHWs in Nigeria is 71,486 (senior = 42,938, junior = 28,548) [11].

The proportion of CHW among healthcare workers in Nigeria is (71,486/347,052 × 100) = 20.6%.

The sample size was calculated using the Leslie Kish formula for estimating minimum sample size for studies: $\:n={Z}^{2}p\left(q\right)/{m}^{2}\:$

Where:

n = the minimum sample size.

Z = the standard normal deviate corresponding to a 95% confidence level.

p = proportion of CHW among healthcare workers in Nigeria is = 20.6% = 0.206.

q = the complementary probability to (1-p) = 1- 0.206 = 0.794.

m = tolerable error margin of 5% = 0.05.

$${\rm{n}} = \frac{{{{(1.96)}^2} \times 0.206 \times (0.794)}}{{{{(0.05)}^2}}} = 251$$

Assuming 10% non-response, the required sample size was increased to 251/0.90 = 280.

### Sampling technique

The mean number of CHW per PHC facility in Nigeria is 8.1 [12, 18]. The total number of PHCs in Kano state − 381, Borno state − 114, Kwara state − 258, Oyo state − 351, and Edo state − 200 [18]. Hence, the total number of CHW in the five states is 10,562.

A multistage sampling method was used.

First stage: A stratified sampling technique was used to proportionately allocate 280 CHW into each of the five states: Kano − 80, Borno − 24, Kwara − 60, Oyo − 74, and Edo – 42. This translated to 10 PHC in Kano, 3 in Borno, 8 in Kwara, 9 in Oyo and 5 in Edo.

Second stage: Simple random sampling was then used to select the number of allocated PHCs in each of the five states.

Third stage: In each PHC, a systematic sampling method was used to recruit 8 CHWs.

Purposive sampling was used to select patients in the selected states for in-depth interviews using the Key Informant Interview Guide.

### Data collection

A semi-structured interviewer-administered questionnaire developed and pre-validated for this study (Appendix I) was used to collect data from all eligible participants who consented to the study. The instrument was validated by sending a draft for evaluation by experts in the fields of cardiology, family medicine and public health. The instrument was also assessed for content and construct validity to ensure coverage of all the areas considered in the study. The reliability of the instrument was determined by administering it to 20 CHWs and subjecting the data collected to Cronbach’s alpha coefficient analysis to determine the internal consistency and reliability of the questionnaire. It was thereafter administered after incorporating all necessary corrections, modifications, and suggestions.

In-depth interviews were conducted for patients using the Key Informant Interview (KII) Guide (Appendix II) to collect data from all the states involved until saturation was achieved. Each session lasted for 45 to 60 min. It was audio-recorded with a moderator and an assistant in attendance who were both trained in the conduct of KII and experts in qualitative studies. The assistant recorded the discussion as well as observed and documented the process for the moderator. Participants were asked open-ended questions on their views of their overall health, and their perceptions on the satisfaction or otherwise of the care received from the CHWs. The study instrument (Appendix I and II) has been uploaded as a supplementary file.

### Data analysis

Information was entered into an Excel spreadsheet and data was analysed using SPSS version 24.0. Qualitative data were recorded and the recordings were transcribed verbatim. Peer checking was done by the researchers where the recordings and transcription were compared to ensure correctness and thereafter the text data was coded using inductive and deductive approaches for the coding process, classified, and presented to team members for evaluation and team discussions until consensus was achieved. To enhance the dependability of the data, the preliminary data, codes, categories, and themes were retained. Thematic analysis of the text data from the KII was done by using Nvivo^®^ version 12 pro which is a qualitative software for data storage, coding, and theme development. Nvivo software was used for transcription of the recorded interview, coding of the responses into themes, determining core themes, systematic team-based coding, creating a Numeric Content Analysis (NCA) table, and preparing the analysed work for publication [19]. Theme development and revision were done iteratively with themes emerging from the data. Member checking was used to secure the credibility of the findings which were then written in summarised form with samples of verbatim quotes provided where necessary.

## Results

**Fig. 1:**
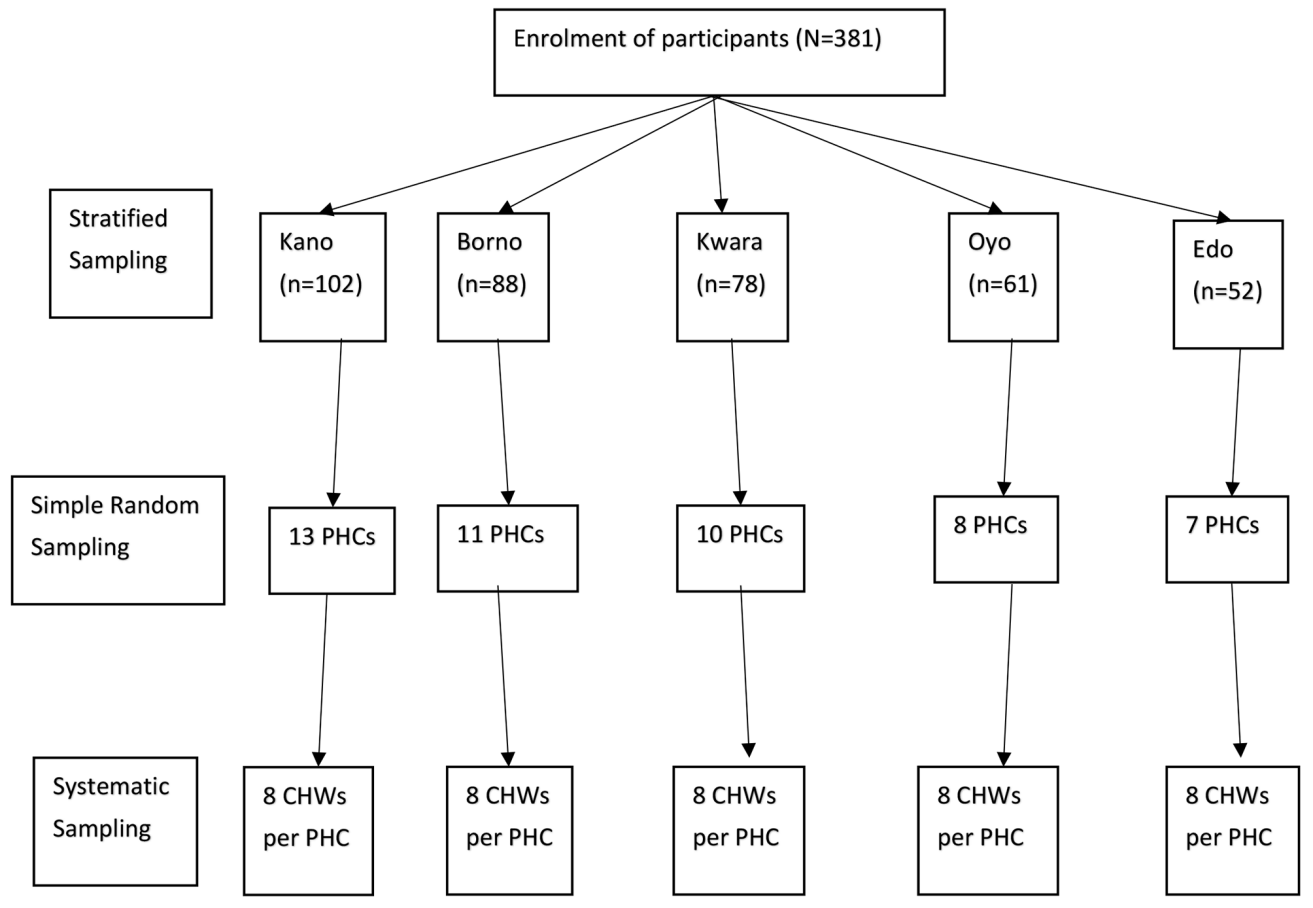
Flow chart of the multi-staged sampling technique used to select CHWs

A total of 381 respondents (Kano – 102, Borno – 88, Kwara – 78, Oyo – 61 and Edo – 52) completed the study (Fig. 1).

Their ages ranged from 19 to 86 years with mean age of 40.96 ± 12.51 years. As shown in Table 1, majority (60.6%) of the respondents belonged to 31–50 years group and are predominantly males (63%) with male to female ratio of 1.7:1. The Senior Community Health Extension Workers (SCHEW) have the highest representation 240 (63%) followed by the Junior Community Health Extension Workers (JCHEW) with 74 (19.4%) and then Community Health Officers with 67 (17.6%). Majority 170(44.6%) had been practicing for > 10 years. The respondents from the North western region had the highest 102 (26.8%), while those in the South-south region had the lowest 52 (13.6%) representation. About a half (50.9%) of the respondents worked under the supervision of doctors, while around three-quarter of them (77.7%) worked under the supervision of nurses in their centres. A higher proportion of the respondents often (29.1%) and always (26.9%) treat patients with hypertension.

**Table 1:** General characteristics of the respondents (*n* = 381)

Variables	Frequency	Percentage
**Age groups (Years)**		
18–30	77	20.2
31–40	128	33.6
41–50	103	27.0
51–60	49	12.9
> 60	24	6.3
**Gender**		
Male	240	63.0
Female	135	35.4
Prefer not to say	6	1.6
**Cadre**		
CHO	67	17.6
JCHEW	74	19.4
SCHEW	240	63.0
**Duration of practice**		
< 1 year	27	7.1
1–5 years	114	29.9
6–10 years	70	18.4
> 10 years	170	44.6
**Region**		
North central	78	20.5
Northeast	88	23.1
Northwest	102	26.8
South-south	52	13.6
Southwest	61	16.0
**Supervised by Doctors**		
No	194	50.9
Yes	187	49.1
**Supervised by Nurses**		
No	85	22.3
Yes	296	77.7
**Treat hypertension**		
Always	102	26.8
Often	111	29.1
Rarely	42	11.0
Sometimes	126	33.1
**Prescribe medications**		
No	18	4.7
Yes	363	95.3

Table 2 shows the respondents’ knowledge on the diagnosis and treatment of hypertension. Headache (89.0%) followed by poor sleep (44.4%) were the commonest symptoms of hypertension known by the respondent, while stroke (75.1%) followed by heart diseases (26.8%) were the commonest complications of hypertension known by them. Using sustained BP ≥ 140/90mmHg as the definition of hypertension, only about one-third of the respondents (31%) could correctly diagnose hypertension. Although, a larger percentage of the respondents (97.9%) counselled patients on lifestyle modifications, only about a half (50.9%) of the respondents request patients to do hypertension-related investigations while only 29.4% actually knew the investigations to be requested. The commonly prescribed medication was Amlodipine (65.6%) and majority (65.1%) of the respondents gave twice weekly follow-up while about half (49.9%) gave monthly follow-up visits. Majority of the respondents referred patients to higher centres due to complications (80.8%), or poor BP control (72.7%). A total of 160 (42.0%) respondents referred patients based on request.

**Table 2:** Knowledge of hypertension management (*n* = 381)

Variables	Frequency	Percentage
**Symptoms** ^**#**^		
Headache	339	89.0
Dizziness	146	38.3
Poor vision	49	12.9
Poor sleep	169	44.4
Palpitation	42	11.0
**Complications** ^**#**^		
Stroke	286	75.1
Heart diseases	102	26.8
Kidney diseases	47	12.3
Death	87	22.8
Eclampsia/convulsion	7	1.8
**Diagnostic skills**		
No	263	69.0
Yes	118	31.0
**Counselling**		
No	8	2.1
Yes	373	97.9
**Request for investigations**		
No	187	49.1
Yes	194	50.9
**Appropriate investigations (n-194)**		
No	137	70.6
Yes	57	29.4
**Prescribed medications** ^**#**^		
Amlodipine	250	65.6
Nifedipine	95	24.9
Lisinopril	110	28.9
Losartan	51	13.4
Methyldopa	98	25.7
Diuretics	118	31.0
B- blockers	27	7.1
**Follow-up** ^**#**^		
Twice weekly	248	65.1
Monthly	190	49.9
Twice monthly	128	33.6
**Reasons for referral** ^**#**^		
Poor BP control	277	72.7
Complications	308	80.8
Patient’s request	160	42.0

#: Multiple Responses

**Fig. 2:**
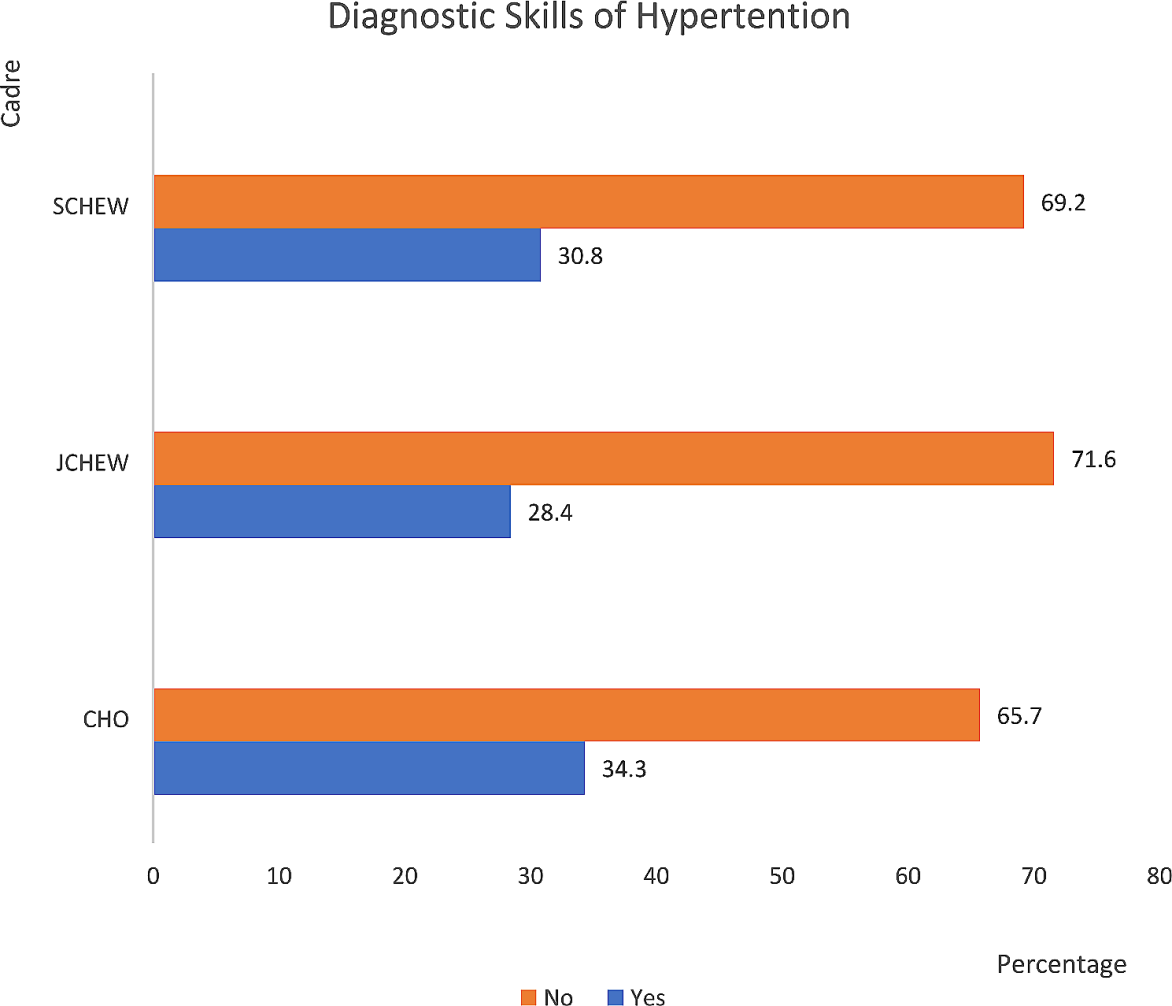
Bar chart showing diagnostic skills of hypertension based on cadre (*n* = 381)

As depicted in Fig. 2, the CHOs have the highest (34.3%) diagnostic skills for hypertension followed by the SCHEWs (30.8%), and then JCHEW (28.4%).

Among the factors that influence the diagnostic skills of the respondents, only gender (FE = 9.205, *p* = 0.008) and geopolitical region (χ^2^ = 20.920, *p* < 0.001) have statistically significant associations with appropriate diagnostic skills for hypertension. The male respondents (40.7%) and those residing in the North western region (47.1%) have the highest percentage of appropriate skills for diagnosing hypertension. Table 3.

**Table 3:** Factors influencing diagnostic skills of hypertension

Variables	No (*n* = 263)	Yes (*n* = 118)	χ^2^	*P* value
**Age groups (Years)**			9.373	0.052
18–30	52(67.5%)	25(32.5%)		
31–40	78(60.9%)	50(39.1%)		
41–50	76(73.8%)	27(26.2%)		
51–60	36(73.5%)	13(26.5%)		
> 60	21(87.5%)	3(12.5%)		
**Gender**			9.205*	**0.008**
Male	80(59.3%)	55(40.7%)		
Female	178(74.2%)	62(25.8%)		
Prefer not to say	5(83.3%)	1(16.7%)		
**Cadre**			0.588	0.745
CHO	44(65.7%)	23(34.3%)		
JCHEW	53(71.6%)	21(28.4%)		
SCHEW	166(69.2%)	74(30.8%)		
**Duration of practice**			2.357	0.502
< 1 year	20(74.1%)	7(29.5%)		
1–5 years	84(73.7%)	30(26.3%)		
6–10 years	46(65.7%)	24(34.3%)		
> 10 years	113(66.5%)	57(33.5%)		
**Region**			20.920	**< 0.001**
North central	55(70.5%)	23(29.5%)		
Northeast	62(70.5%)	26(29.5%)		
Northwest	54(52.9%)	48(47.1%)		
South-south	41(78.8%)	11(21.2%)		
Southwest	51(83.6%)	10(16.4%)		
**Supervised by Doctors**			0.041	0.839
No	133(68.6%)	61(31.4%)		
Yes	130(69.5%)	57(30.5%)		
**Supervised by Nurses**			2.009	0.156
No	64(75.3%)	21(24.7%)		
Yes	199(67.2%)	97(32.8%)		

**Bold**: Statistically significant * Fisher’s Exact Test

In addition, age (χ^2^ = 13.249, *p* = 0.010), gender (FE = 11.514, *p* = 0.003), region (χ^2^ = 30.485, *p* < 0.001) and being supervised by doctors (χ^2^ = 5.534, *p* = 0.019) are significantly associated with appropriate knowledge of baseline investigations for hypertension. Respondents within the age group of 31–50 years (68.9%), male (37.1%) and unidentified (100%) gender, those residing in the North western region (54.9%), and those under doctors’ supervision (36.5%) have the highest percentage for appropriate knowledge of baseline investigations. Table 4.

**Table 4:** Factors influencing knowledge of appropriate investigations for hypertension

Variables	No (*n* = 137)	Yes (*n* = 57)	χ^2^	*P* value
**Age groups (Years)**			13.249	**0.010**
18–30	28(90.3%)	3(9.7%)		
31–40	45(66.2%)	23(33.8%)		
41–50	37(64.9%)	20(35.1%)		
51–60	19(86.4%)	3(13.6%)		
> 60	8(50.0%)	8(50.0%)		
**Gender**			11.514*	**0.003**
Male	44(62.9%)	26(37.1%)		
Female	93(76.9%)	28(23.1%)		
Prefer not to say	0(0.0%)	3(100.0%)		
**Cadre**			3.429	0.180
CHO	31(66.0%)	16(34.0%)		
JCHEW	22(61.1%)	14(38.9%)		
SCHEW	84(75.7%)	27(24.3%)		
**Duration of practice**			0.466	0.926
< 1 year	7(70.0%)	3(30.0%)		
1–5 years	37(74.0%)	13(26.0%)		
6–10 years	25(71.4%)	10(28.6%)		
> 10 years	68(68.7%)	31(31.3%)		
**Region**			30.485	**< 0.001**
North central	32(91.4%)	3(8.6%)		
Northeast	31(75.6%)	10(24.4%)		
Northwest	23(45.1%)	28(54.9%)		
South-south	14(58.3%)	10(41.7%)		
Southwest	37(86.0%)	6(14.0%)		
**Supervised by Doctors**			5.534	**0.019**
No	71(78.9%)	19(21.1%)		
Yes	66(63.5%)	38(36.5%)		
**Supervised by Nurses**			1.383	0.240
No	32(78.0%)	9(22.0%)		
Yes	105(68.6%)	48(31.4%)		

**Bold**: Statistically significant * Fisher’s Exact Test

### Key informant interviews

Key informant interviews were conducted for 70 patients with hypertension across the five states (Kano – 20, Borno – 15, Kwara – 15, Oyo – 10 and Edo – 10). However, saturation was achieved after analysing data for 14 patients across the 5 states (Kano – 4, Borno – 3, Kwara – 3, Oyo – 2 and Edo – 2) and the results are presented below. The remaining 56 were discarded as no new information was obtained from repeated analysis of the interview data. The sociodemographic characteristics are summarized in Table 5. They were mostly females (71.4%), in their 50s (50.0%) with a mean age of 53.5 ± 7.54 years and have mostly been hypertensive for 1 to 5 years (57.1%) with a mean duration of 8.25 ± 7.45 years. Respondents were predominantly traders (35.7%) with secondary level of education (35.7%). They were mostly Hausa/Fulani (42.3%).

**Table 5:** Sociodemographic Characteristics of Participants in the KII *N* = 14

Factors	Frequency	Percentage
**Age**		
< 50	5	35.7
50–59	7	50.0
≥ 60	2	14.3
Mean ± SD; Min, Max 53.5 ± 7.54; 43, 73		
**Gender**		
Male	4	28.6
Female	10	71.4
**Duration of Hypertension (Years)**		
1–5	8	57.1
6–10	2	14.3
> 10	4	28.6
Mean ± SD; Min, Max 8.25 ± 7.45; 1, 27		
**Level of Education**		
Qur’anic	2	14.3
Primary	4	28.6
Secondary	5	35.7
Tertiary	3	21.4
**Occupation**		
House Wife	4	28.6
Farmer	3	21.4
Trader	5	35.7
Civil Servant	2	14.3
**Tribe**		
Hausa/Fulani	6	42.9
Yoruba	5	35.7
Edo	2	14.3
Kanuri	1	7.1

### Contributory role of community health workers to the management of hypertension

The participants acknowledged receiving both counselling and medications for their hypertension from a CHW. Some were diagnosed by the CHWs and have been receiving treatment from them ever since while others were diagnosed in a secondary or tertiary health facility but go the PHC for drug refill by the CHW and BP check from time to time. A 73 year old housewife from Kano State who has been hypertensive for 27 years said:

*I receive counselling and medication from the Community Health Extension Workers (CHEWs) in this PHC since I was diagnosed of hypertension 27 years ago. They check my BP*,* give me medications*,* advise me on what to eat and tell me when to come for check-up*.

A 49 year old farmer who has been hypertensive for 9 years said:

*I was diagnosed at the general hospital where I go for check-up. I however come to the health centre for routine drugs and counselling from the health centre*,* especially if I do not have a serious complain or I don’t have enough money to go to the hospital*.

### Satisfaction with service of community health workers managing hypertension

Overall, the patients reported positive experiences and satisfaction with the services provided, suggesting a positive contribution of satisfactory services by community health workers to the management of hypertension within the community.

A 49 year old female civil servant who has been hypertensive for 12 years said:

“*I rate the treatment received here highly and I am satisfied with the services provided by the CHEW*”.

According to a 53-year-old male farmer from Edo State:

“*Despite some dissatisfaction with waiting times*,* overall*,* I am satisfied with the services provided by the health workers. They listen to you*,* counsel you and give you drugs after checking your BP*”.

## Discussions

Community health workers (CHWs) constitute a major work force in the healthcare sector especially in primary health care centres (PHCs) [9, 20]. Our findings provide valuable insights into various characteristics of CHWs including their knowledge and practices in the management of hypertension among patients presenting to their facility.

Senior Community Health Extension Workers (SCHEW) constituted the largest proportion of respondents, followed by Junior Community Health Extension Workers (JCHEW) and Community Health Officers. This distribution reflects the hierarchical structure within the community health sector and underscores the significant role played by community health extension workers in healthcare delivery, particularly in underserved areas.

In terms of clinical practice, a considerable proportion of CHWs reported frequently treating patients with hypertension. This finding underscores the crucial role of CHWs in the prevention, early detection, and management of non-communicable diseases, such as hypertension, within community settings [9, 20, 21].

The high proportion of CHWs working under the supervision of nurses compared to doctors, highlights the vital role of nurses in primary healthcare settings, where they often serve as frontline healthcare providers and bridge the gaps of insufficient numbers of doctors [7]. Strengthening the capacity of nurses and other allied healthcare professionals through training and capacity-building initiatives may enhance the delivery of comprehensive primary healthcare services and improve health outcomes in the communities served by the CHWs [7].

The recognition of symptoms and complications associated with hypertension among the CHWs is crucial for early detection and management. It is noteworthy that headache was identified as the most known symptom of hypertension, followed by poor sleep. Similarly, stroke emerged as the most recognized complication of hypertension, indicating a reasonable level of awareness among the participants regarding the potential health risks associated with uncontrolled hypertension.

However, despite the relatively high awareness of symptoms and complications, the correct diagnosis of hypertension remains a challenge, with only about one-third of the participants able to correctly diagnose hypertension using the standard criteria of sustained blood pressure (BP) ≥ 140/90mmHg. This is consistent with another study conducted among healthcare professions to determine the knowledge of blood pressure measurement skills, where one in five persons measured blood pressure correctly [22]. It is also consistent with studies conducted among nurses which revealed irregularities in the blood pressure assessment technique at baseline evaluation which is due to the fact that there was inadequate emphasis on correct technique and lack of periodical retraining [23].

Results from our study contradict what was reported in a community based hypertension screening study performed by health extension workers and trained health professionals where high blood pressure was detected in a dependable way using an aneroid sphygmomanometer [24]. This could be attributed to the fact that an intensive training was given to them and adequate supervision during blood pressure assessments. Baseline assessment from the study conducted by Check et al. revealed that majority of the clinical skills carried out by an average provider were not performed accurately and it indicated that blood pressure assessment knowledge and clinical skills decline over time which leads into technical errors and incorrect readings [25]. This highlight significant gaps in knowledge and management of hypertension among the CHEWs, which may impede timely identification and management of hypertension in clinical practice [26].

In terms of management, majority of the respondents counselled patients on lifestyle modification, prescribe medications and book patients for follow up visit. In contrast, small percentage of the participants were familiar with hypertension related investigations, which underscore the need for targeted educational interventions to enhance the clinical competence of community health workers in the diagnosis and management of hypertension [26].

However, this study revealed that age group of 31–50 years, male gender, practicing in north western region and being supervised by doctors are factors that are significantly associated with appropriate diagnostic skills for hypertension. The result is similar to findings from a study conducted among tertiary hospital staff to determine the knowledge of blood pressure measurement and its related socio demographic determinants, which revealed that a significant proportion of male hospital staff had a good knowledge of blood pressure measurement than females [27]. The higher knowledge of hypertension care by CHWs in the North western region (Kano) could be due to the rolled out of the National Hypertension Control Initiative (funded by an NGO called Resolve to Save Lives – RTSL, in collaboration with FMoH, WHO and NPHCDA) in Kano and Ogun state since 2020. As at 2022, the initiative has trained primary healthcare providers in 104 PHC in both states.

Also, this study revealed that age group of 31–50 years, male sex, region and being supervised by doctors were significantly associated with knowledge of baseline investigations for hypertension. This is similar to findings from a study conducted by Mbekwa et al. where male sex, age less than 63 years and higher level of education were significantly associated with the knowledge of hypertension [28].

This contradicts results from another study which shows that there was no association between sex or educational level and knowledge about hypertension but a relationship existed between the age group below 30 years and a lower level of knowledge than the other age groups [29]. A study conducted in Malaysia identified female gender, age, race, location and educational level as significant factors associated with knowledge about hypertension [30]. The study suggested that females pay more attention to their health than male.

Patients presenting to CHWs for their hypertension management expressed satisfaction in the care received. This was evident in the KII conducted for hypertensives receiving care from CHW. This underscores the need to train CHWs to be able to identify and treat uncomplicated hypertension as well as refer patients with poorly controlled hypertension and those with complications as appropriate.

### Limitations

The study relied on self-report on services rendered by CHWs. This could lead to potential biases in self-reported data.

Five states out of the thirty-six states and Federal Capital Territory (FCT) were studied. This calls to question concerning the representativeness of the samples. However, the states were drawn from five of the six geopolitical zones in Nigeria giving the study a national spread.

## Conclusion

In conclusion, this study underscores the significant contribution of CHWs to hypertension management in Nigeria. Their pivotal roles encompass diagnosing hypertension, treating and following up hypertensive patients, as well as providing counselling. Overall, patients expressed satisfaction with the care provided by CHWs. Nonetheless, enhancing their effectiveness and ensuring optimal health outcomes for hypertensive patients requires comprehensive training and regular supervision of these CHWs.

### Recommendations

There is need to formally involve CHWs in the management of hypertension in Nigeria and elsewhere. Patients could go to PHC where CHWs could check their BPs, refill their drugs and counsel them on appropriate lifestyles to control hypertension.

Government should train CHWs across the PHC nationwide on accurate diagnosis and management of hypertension including prompt referral to hospitals with trained physicians when the need arises.

Government should employ physicians in the PHC across the country to supervise the CHWs. This will improve their management skills and reduce the burden of hypertension in Nigeria.

### Electronic supplementary material

Below is the link to the electronic supplementary material.

Supplementary Material 1

## Data Availability

All data generated or analysed during this study are included in this published article.
