# Supplementary material for: The role of community health workers in the management of hypertension in Nigeria
Source: BMC Prim Care. 2024 Jul 20;25:266. doi: 10.1186/s12875-024-02521-2 (PMC11265034; doi:10.1186/s12875-024-02521-2)
Supplement: Supplementary file 1 — Supplementary Material 1 [file 12875_2024_2521_MOESM1_ESM.docx]

**APPENDIX I: QUESTIONNAIRE**

**Role of community of community health workers in the management of hypertension in Nigeria**

**CHWs**

| Study ID |  |
| --- | --- |
| Location | NE [1] NW [2] NC [3] SS [4] SE [5] SW [6] |
| State |  |
| Hospital |  |
| Initial |  |
| Age (Years) |  |
| Gender | Male [1] Female [2] |
| Cadre | CHO [1] SCHEW [2] JCHEW [3] |
| Year of practice | Less than 1 [1] 1 to 5 years [2] 5 to 10 years [3] greater than 10 years [4] |
| Do you have a Doctor at your centre of practice? | Yes [1] No [2] |
| Do you have a Nurse at your centre of practice? | Yes [1] No [2] |
| How often do you as a CHW treat patients with hypertension? | Always [1] Often [2] Sometimes [3] Rarely [4] Never [5] |
| Diagnosis of hypertension (state the blood pressure) | SBP [ ]  DBP [ ] |
| Do you request for laboratory investigation after diagnosis | Yes [1] No [2] |
| If yes to above (list the investigation) |  |
| Do you counsel patients with hypertension on its management? | Yes [1] No [2] |
| Do you prescribe drug(s) for the treatment of hypertension? | Yes [1] No [2] |
| kindly list the drug(s) you prescribe. |  |
| List the common symptom(s) of hypertension here. |  |
| Kindly list the complication(s) of hypertension. |  |
| How often do u follow up patients with hypertension? | Weekly [1] Fortnightly [2] Monthly [3] Once in two months [4] Once in three months [5] Quarterly [6] Only when patient have complaints [7] |
| Do you refer patients with hypertension to secondary/tertiary centres | Yes [1] No [2] |
| At what point do you refer patients | Development of complications [1] poor control [2] others [3]  Others specify………………………………….. |

**APPENDIX II: Key informant interview guide**

**Role of CHW in the Management of Hypertension**

Interview details

Participant code: ______

Interviewer’s name: _______

Location of participant: _____________

Platform used to conduct the interview (face-to-face, zoom, WhatsApp, etc): __________

Date of interview (DD/MM/YYYY):____________________

General Instructions: Most questions below have probes that are intended to be follow-up questions. Please be sure to ask the question and allow the participant to respond before asking additional questions. Please adjust the order and pacing as needed.

Interviewer, please start with an introduction such as:

Thank you for taking the time to meet with me today. I would like to ask you some questions about your experience in the PHC where you access care for your hypertension.

The interview should take no more than 30-45 minutes. (General purpose of the interview)

I will also be interviewing other patients Your cooperation is important because it will help improve hypertension management in PHCs. (Why your cooperation is needed).

I would like to record the conversation to make sure I don’t miss any of your comments. I may also take some notes during the interview. All your responses will be kept confidential within the study team. We will also ensure that any information we include in our report does not identify you as the respondent. Additionally, only the team will know the code I assign you today, so others will not know what we discuss today. The information you provide will potentially identify areas for improvement. This should lead to improved outcome for patients with hypertension accessing care in PHCs. (What will happen with the collected information and how the community will benefit.)

You don’t have to talk about anything you don’t want to and you may end the interview at any time. We appreciate you answering these questions as honestly as possible. Please feel free to ask me if you have any questions.

Do you consent to participate in this interview? Y____ N____

If the participant consents and answers yes, begin. Stop to answer any questions people may have before starting.

Do you consent to have this interview recorded? Y____ N_____

If participant consents and answers yes, begin recording and ask the previous two questions again, under the recording.

Proceed with the interview.

1. I will start by introducing myself. I am Dr …………..
2. Now tell me about yourself.
   1. How old are you?
   2. What’s your gender?
   3. What’s your occupation?
   4. What is your highest qualification?
   5. What tribe are you from?
3. Tell me about your hypertension – When where you diagnosed, where have you been receiving treatment, etc.
4. In your own words, what is your experience like accessing care in a PHC facility?
   1. When did you start accessing care in a PHC?
   2. Who is attending to you there – CHW, CHEW, JCHEW, don’t know.
   3. Can you list what they do for you when you come?
5. What has been your experience with the care given?

Probe further:

- 1. How has it been beneficial?
  2. How has it been unhelpful?

If necessary, probe further:

c. What negative experience have you had with the PHC?

d. What positive experience do you remember?

1. What medications are you currently on and who prescribed them?

1. Is your blood pressure controlled to your satisfaction? Explain.
2. Have you ever been referred by the CHW to a secondary or tertiary health facility?
   1. If yes, what was the reason for the referral?
   2. If no, why do you think they have not referred you?
3. Would you prefer to be seen and managed in a health centre by a CHW for your hypertension or by a nurse or a doctor? Explain your preference.
4. In what ways do you think PHCs could be improved to better manage patients with hypertension?

Probe further:

- 1. What recommendations do you have to address the problems you see in the PHC facility?
  2. Would you recommend the PHC to any of your friends/families/colleagues?

1. Today you have shared with me about ____, ______ and ______. Before we conclude this interview, is there anything else you’d like to share that would be important for us to know about your experience with the care received in PHC?

This is the end of the interview. Thank you for your time!
